# Association between the *ENPP1* K121Q Polymorphism and Risk of Diabetic Kidney Disease: A Systematic Review and Meta-Analysis

**DOI:** 10.1371/journal.pone.0118416

**Published:** 2015-03-20

**Authors:** Denise Alves Sortica, Marjorie Piucco Buffon, Bianca Marmontel Souza, Bruna Bellicanta Nicoletto, Andressa Santer, Tais Silveira Assmann, Daisy Crispim, Luis Henrique Canani

**Affiliations:** 1 Endocrine Division, Hospital de Clínicas de Porto Alegre, Porto Alegre, RS, Brazil; 2 Postgraduate Program in Medical Sciences: Endocrinology, Universidade Federal do Rio Grande do Sul, Porto Alegre, RS, Brazil; University of Milan, ITALY

## Abstract

The potential association between the K121Q (A/C, rs1044498) polymorphism in the ectonucleotide pyrophosphatase/phosphodiesterase (*ENPP1*) gene and risk of diabetic kidney disease (DKD) has been investigated. Nevertheless, the effect of this variant on DKD risk is still under debate, and conflicting results have been reported. To this date, no meta-analysis has evaluated the association of the K121Q polymorphism with DKD. This paper describes the first meta-analysis conducted to evaluate whether the *ENPP1*K121Q polymorphism is associated with DKD. A literature search was conducted to identify all case-control or cross-sectional studies that evaluated associations between the *ENPP1*K121Q polymorphism and DKD. Pooled odds ratios (OR) and 95% confidence intervals (95% CI) were calculated for allele contrast, additive, dominant and recessive inheritance models. Seven studies were eligible for inclusion in the meta-analysis, providing data on 3571 type 1 or type 2 diabetic patients (1606 cases with DKD and 1965 diabetic controls without this complication). No significant heterogeneity was observed among the studies included in the meta-analysis when assuming different inheritance models (I^²^ < 50% or P > 0.10 for the entire sample and after stratification by ethnicity). Meta-analysis results revealed significant associations between the K121Q polymorphism and risk of DKD in Asians and Europeans when assuming the different inheritance models analyzed. The most powerful association was observed for the additive model (OR = 1.74, 95% CI 1.27-2.38 for the total sample). In conclusion, the present meta-analysis detected a significant association between the *ENPP1*K121Q polymorphism and increased susceptibility of DKD in European and Asian populations.

## Introduction

The number of people living with diabetes mellitus (DM) worldwide is expected to double between 2000 and 2030 [1,2]. This global increase in the prevalence of DM will lead to a higher incidence of the chronic complications of diabetes [3]. Diabetic kidney disease (DKD) is a common diabetic chronic complication [4]. DKD is characterized by albuminuria and reduced glomerular filtration rate (GFR) [4]. Approximately 40% of patients with diabetes, whether type 1 or type 2, develop some degree of renal disease after many years of DM [5]. DKD is thus the most common cause of end-stage renal disease in several countries, and it is associated with high morbidity and mortality rates among diabetic patients [6,7].

The mechanisms involved in the pathogenesis of DKD are multiple and complex. Both epidemiological and familial studies have shown major agreement for clustering of DKD in some families, strengthening the hypothesis that there are important genetic factors involved in its pathogenesis [7]. Environmental factors such as hyperglycemia, arterial hypertension and/or dyslipidemia are known to play a key role in the development of DKD in genetically susceptible subjects [5,6,8,9]. Therefore, extensive efforts have been made to identify which genes are related to DKD; however, results are still inconclusive due to several genes being associated with small effects in different populations [10,11,12,13]. The identification of such genes will help detect individuals at high risk of developing DKD, and might provide a better understanding of its pathophysiology [14].

Candidate genes for insulin resistance (IR) can also be considered as DKD candidate genes in patients with type 1 or type 2 DM [15]. In this context, the gene that encodes the ectonucleotide pyrophosphatase/phosphodiesterase (ENPP1) is a good candidate gene for DKD [12,16]. The *ENPP1* gene is expressed in many tissues, including the kidneys, and it is known that increased *ENPP1* gene expression blockades the tyrosine kinase activity of the insulin receptor in several cells, causing IR [7]. More than 15 years ago, a polymorphism was reported in exon 4 of this gene leading to a lysine (K) to glutamine (Q) substitution in codon 121 (K121Q; rs1044498) [7,12]. This change is located in one of the ENPP1 somatomedin-B-like domain, and might influence protein-protein interactions [7,17]. Furthermore, the Q allele of the K121Q variant has been shown to influence ENPP1 protein function by inhibiting insulin receptor function and insulin signaling more effectively than the K allele [12].

Since then, several studies have investigated the association between the K121Q polymorphism and IR, type 2 DM (T2DM) and/or its chronic complications, such as DKD (for a review, see [7]). However, the impact of this polymorphism on DKD susceptibility is still under debate, with contradictory findings: while some studies reported an association of the Q allele with DKD [18,19,20,21], other studies were not able to replicate this association [22,23].

Thus, to further investigate the potential association of the *ENPP1* K121Q polymorphisms with DKD, we conducted a systematic review and meta-analysis of the literature on the subject.

## Materials and Methods

### Search strategy and eligibility criteria

This study was designed and reported in agreement with the Preferred Reporting Items for Systematic Reviews and Meta-Analyses (PRISMA), Meta-analysis of Observational Studies in Epidemiology (MOOSE) statements, and following the Meta-analysis on Genetic Association Studies Checklist from Plos One (**S1 Table**) [24,25].

PubMed and Embase databases were searched systematically to identify all genetic studies that investigated associations between DKD and the K121Q polymorphism. The K121Q polymorphism was selected for the present meta-analysis because it has been the most frequently studied polymorphism in *ENPP1* gene. There are not enough data concerning the other *ENPP1* polymorphisms and diabetic kidney disease to perform a meta-analysis. The following medical subject headings (MeSH) were used: ("Phosphodiesterase I"[7] OR "ectonucleotide pyrophosphatase phosphodiesterase 1"[Supplementary Concept]) AND ("Polymorphism, Genetic"[Mesh] OR "Polymorphism, Single Nucleotide"[Mesh] OR "Polymorphism, Restriction Fragment Length"[Mesh] OR "Amplified Fragment Length Polymorphism Analysis"[Mesh] OR "Polymorphism, Single-Stranded Conformational"[Mesh] OR "DNA Copy Number Variations"[Mesh] OR "Mutation"[Mesh] OR "Mutation, Missense"[Mesh] OR "INDEL Mutation"[Mesh] OR "Point Mutation"[Mesh] OR "Frameshift Mutation"[Mesh] OR "Codon, Nonsense"[Mesh]) AND ("Diabetes Mellitus"[Mesh] OR "Diabetes Complications"[Mesh] OR "Diabetes Mellitus, Type 2"[Mesh] OR "Diabetes Mellitus, Type 1"[Mesh]). The search was restricted to papers reporting on human subjects and published in English or Spanish, and was completed on July 25th, 2014. It is worth noting that although we aimed to analyze only studies published in English or Spanish, we did not identify any study in another language which analyzed the K121Q polymorphism and diabetic kidney disease. All of the papers found were also searched manually to identify other relevant citations. Moreover, unpublished results were searched in the abstract books of the Endocrine Society, American Diabetes Association, and European Association for the Study of Diabetes (EASD) Meetings.

Eligibility was evaluated through a review of titles and abstracts; when abstracts did not provide sufficient information, the full text of the paper was retrieved for analysis, as in previous reviews by our group [26,27,28]. Briefly, this was done independently in a standardized manner by two investigators (D.A.S and M.P.B.). Disagreements were resolved by discussion between them and, if required, a third reviewer (D.C.) was consulted. We included observational studies that evaluated the frequencies of the K121Q polymorphism in patients with DKD (cases) and diabetic patients without any degree of DKD (controls). Both type 1 and type 2 diabetic patients older than 18 years were included. Studies would be excluded from analysis if the genotype distributions in the control group deviated from those predicted by Hardy-Weinberg equilibrium (HWE) or if they did not provide sufficient data to estimate an odds ratio (OR) with 95% CI. However, no study was excluded due to these criteria. If results were duplicated and had been published more than once, the most complete article was included in the study.

### Data extraction

Necessary information from each study was extracted by two investigators working independently (D.A.S. and M.P.B.), using a standardized extraction form, and consensus was sought for all extracted items. When consensus could not be achieved, differences in data extraction were decided by reading the original publication [26,27,28]. The data extracted from each study were as follows: (1) characteristics of the study (including name of the first author, year of publication, number of subjects included in the case and control groups) and sample characteristics, such as age, gender, ethnicity, type of DM, DM duration, HbA1c, body mass index (BMI), systolic and diastolic blood pressure, percentage of hypertension, lipid profile, DKD classification and information regarding kidney function; (2) case and control definitions; (3) polymorphism frequencies (including genotype and allele distributions in case and control groups and ORs with 95% CIs). When data were not available, the authors were contacted by email.

### Quality control assessment

Two investigators (D.A.S. and D.C.) independently evaluated the quality of each eligible article using the Newcastle-Ottawa Scale (NOS) for observational studies [29]. The NOS contains nine items subdivided into three dimensions, including selection, comparability, and exposure. For each item, a sequence of answer alternatives is provided. A star scoring system is used for semi-quantitative evaluation of article quality, such that the highest-quality studies are assigned a maximum of one star for each item, with the exception of the comparability item, which can be assigned two stars. Therefore, the total NOS score ranges from zero to nine stars.

### Statistical analysis

Control subjects’ genotype frequencies were tested for compliance with HWE using a goodness-of-fit chi-square (χ^2^) test. Gene-disease associations were measured using OR (95% CI) estimations based on the following genetic inheritance models: 1) allele contrast; 2) additive model; 3) recessive model; 4) and dominant model [30]. Taking into account that the frequency of the *ENPP1* K121Q polymorphism varies across different populations, gene-disease associations for the different inheritance models were also analyzed according to ethnicity.

Heterogeneity was evaluated using a χ^2^-based Cochran’s Q statistic and inconsistency was tested by the I^2^ metric. Heterogeneity would be considered statistically significant at P <0.10 for the Q statistic or I^2^ >50% for the I^2^ metric statistic (29, 30). However, since no significant heterogeneity was detected, the fixed effect model (FEM) was used to calculate OR (95% CI) for each individual study and for the pooled effect [31]. Due to the lack of inter-study heterogeneity, we did not perform meta-regression analyses (adjusting for covariables such as age, sex, body mass index or environmental factors) or sensitivity analysis. All statistical analyses were performed using Stata 11.0 software (StataCorp, College Station, TX, USA).

## Results

**Fig 1:**
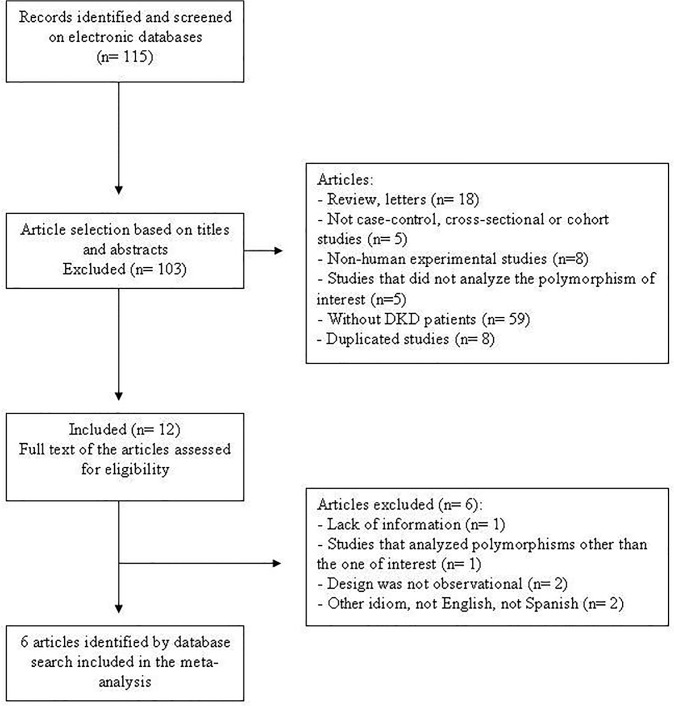
Flowchart illustrating the search strategy used to identify studies of association between the *ENPP1* K121Q polymorphism and diabetic kidney disease for inclusion in the meta-analysis.

Our search strategy yielded 115 possibly relevant papers (**Fig. 1**), 103 of which were excluded following the review of titles and abstracts. Twelve articles appeared eligible after this phase and were selected for full-text evaluation. Nevertheless, after cautious reading of the full texts, further studies were excluded owing to missing information, ineligible study design or because the *ENPP1* polymorphism reported was not the one of interest for this meta-analysis. Therefore, seven articles fulfilled the eligibility criteria and were included in the meta-analysis, providing data on 3571 subjects (1606 cases with DKD and 1965 diabetic controls without this complication). The study reported by Leitão *et al*. [10] was subdivided into two studies because it analyzed the *ENPP1* polymorphism in two different populations.

**S2 Table** depicts the main characteristics of the studies included in our meta-analysis. Regarding the DKD classification, Canani *et al*. [18] subdivided cases into two groups, proteinuric and end-stage renal disease (ESRD), while Leitão *et al*. [10] and De Cosmo *et al*. [20] defined DKD patients as microalbuminuric or macroalbuminuric. Wu *et al*. [19], Lin *et al*. [21] and Tarnow *et al*. [32] classified all DKD cases as macroalbuminuric. **S3 Table** lists genotype and allele distributions and ORs (95% CI) for the *ENPP1* K121Q polymorphism in case and control groups from the five articles reviewed.

A quality evaluation of each individual study included in the meta-analysis is shown in **Table 1**. The highest-quality papers were awarded nine stars. Overall, most studies were classified as having at least moderate quality in terms of selection, comparability and exposure criteria. Wu *et al*. [19] was awarded eight stars; Lin *et al*. [21], Canani *et al*. [18] and De Cosmo *et al*. [20] were awarded seven stars; Tarnow *et al*. [32], six stars; and Leitão *et al*. [10], five stars.

**Table 1:** Newcastle-Ottawa quality assessment scores for the studies included in the meta-analysis.

Author	Year	Selection	Comparability	Exposure
Tarnow et al. [32]	2001	**	**	**
Canani et al. [18]	2002	***	*	***
Leitão et al. [10]	2008	**		***
De Cosmo et al. [20]	2009	***	**	**
Wu et al. [19]	2009	***	**	***
Lin et al. [21]	2011	**	**	***

**Fig 2:**
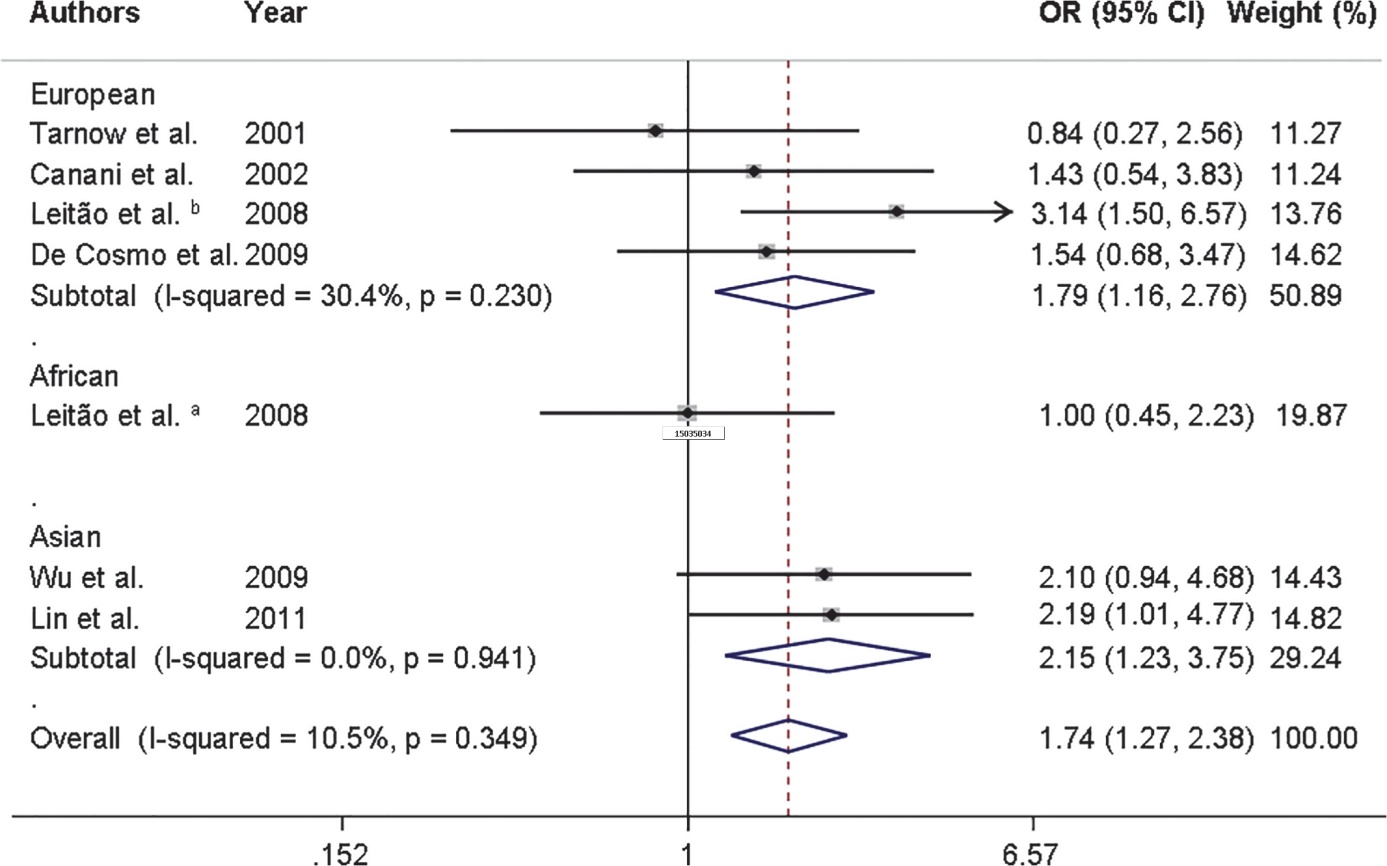
Forest plot showing individual and pooled ORs (95%CI) for the association between the *ENPP1* K121Q polymorphism and diabetic kidney disease after stratification by ethnicity, under an additive inheritance model. The area of the squares reflects the study-specific weight. The diamond illustrates the fixed-effect model summary OR (95% CI).

**Table 2** summarizes the results of the pooled meta-analyses for associations between the *ENPP1* K121Q polymorphism and risk of DKD. In general, our results revealed significant associations between the K121Q polymorphism and risk of DKD when assuming allele contrast, additive, recessive and dominant inheritance models. Notably, the most powerful association was observed for the additive model (OR = 1.74, 95% CI 1.27–2.38). Moreover, after stratification by ethnicity, the associations between the K121Q polymorphism and risk of DKD remained in Europeans and Asians (**Table 2 and Fig. 2**). There was only one study performed in Africans [10], which showed that the K121Q polymorphism was not associated with DKD in this population.

**Table 2:** Pooled measures for association between the *ENPP1* K121Q polymorphism and susceptibility to diabetic kidney disease.

Inheritance model	No. of studies	No. of cases	I^2^ (%)	Pooled OR (95% CI)
**Allele contrast**				
Overall*	7	1,606	41.0	1.36 (1.20–1.53)
European	4	1,102	17.9	1.28 (1.11–1.49)
Asian	2	431	0.0	1.72 (1.35–2.18)
				
**Additive**				
Overall*	7	1,606	10.5	1.74 (1.27–2.38)
European	4	1,102	30.4	1.79 (1.16–2.76)
Asian	2	431	0.0	2.15 (1.23–3.75)
				
**Recessive**				
Overall*	7	1,606	5.5	1.54 (1.15–2.08)
European	4	1,102	31.8	1.71 (1.11–2.62)
Asian	2	431	0.0	1.75 (1.01–3.02)
				
**Dominant**				
Overall*	7	1,606	43.7	1.40 (1.21–1.62)
European	4	1,102	27.2	1.28 (1.08–1.52)
Asian	2	431	0.0	1.91 (1.43–2.55)

No significant heterogeneity was observed among the analyzed studies investigating the *ENPP1* K121Q polymorphism when assuming different inheritance models. Thus, fixed effect models (FEM) were used for the calculation of OR (95% CI) for each individual study and for the pooled effect.

* Studies included: 4 with patients of European descent; 2 with patients of Asian descent; 1 with patients of African descent.

No significant heterogeneity was observed among the analyzed studies investigating the *ENPP1* K121Q polymorphism when assuming different inheritance models (I^2^ < 50% or P > 0.10 for the entire sample and also after stratification by ethnicity; **Table 2**).

## Discussion

The effect of the *ENPP1* K121Q polymorphism on susceptibility to DKD is still controversial: while some studies reported an association of the Q allele with DKD risk [18,19,20,21,33], other studies were not able to replicate this association [22,23], possibly due to small sample sizes and differences in K121Q frequencies among ethnicities. Therefore, to further evaluate whether this polymorphism is associated with DKD risk, we conducted a meta-analysis of five studies carried out in different populations. Our results revealed significant associations between the K121Q polymorphism and risk of DKD in all genetic inheritance models analyzed.

As already mentioned, increased ENPP1 levels blockade the tyrosine kinase activity of the insulin receptor α-subunit in many cells, causing IR [7,34,35]. Consequently, ENPP1 has being associated with impaired glucose metabolism. Subjects with IR have high concentrations of ENPP1 protein [7,36,37,38,39]. Accordingly, transgenic mice overexpressing *ENPP1* in skeletal muscles and liver showed elevated glucose and insulin concentrations as well as decreased glucose uptake in muscle [40]. In contrast, the total knockdown of *ENPP1* gene in the mice liver was able to decrease both postprandial and fasting plasma glucose levels [7,41].

Thus, taking into account the role of ENPP1 in IR, several studies have investigated the association between the *ENPP1* K121Q polymorphism and IR or T2DM (reviewed in [7]). To this date, eight meta-analyses had reported that *ENPP1* 121Q allele carriers in different populations and ethnicities are at increased risk of developing T2DM [11,42,43,44,45,46,47,48]. In contrast, meta-analyses performed by Zhao *et al*. [49] and Weedon *et al*. [50] did not find such an association with T2DM in Europeans.

Regarding the association between the K121Q polymorphism and DKD or related features, De Cosmo *et al*. [51] showed that this variant influences GFR decline in proteinuric type 1 diabetic patients: GFR decreased earlier in subjects with QQ/KQ genotypes compared to subjects with KK genotype. We previously demonstrated that the Q variant was associated with ESRD in type 1 diabetic patients and short DM duration [18]. Q allele carriers (KQ/QQ) had a two fold increased risk of developing ESRD when compared to patients with KK genotype (OR 2.3; 95% CI 1.2–4.6) [18]. Moreover, De Cosmo *et al*. [20] reported that Italian T2DM patients with the Q allele had an increased risk of having a decreased GFR as well as more severe DKD than non-carriers. This is in accordance with data reported by Lin *et al*. [21] and Wu *et al*. [19], who also described an association between the Q allele and increased risk of DKD in Asian patients with T2DM. Nevertheless, other studies did not observe any association between the K121Q polymorphism and DKD or related features [7,10,23,32]. This might be due to an effect of ethnicity on this association. The present meta-analysis suggests that the Q allele is significantly associated with DKD in both Europeans and Asians, possibly under an additive inheritance model. There was only one [10] study that included African descent subjects. Therefore, the role of this polymorphism in these subjects remains to be determined. Taking into account the small number of studies carried out in each ethnicity, additional studies with larger sample sizes are still needed to confirm the association between the K121Q variant and DKD in different ethnicities.

It is worth noting that several genome wide association studies have searched for chromosomal regions linked or associated with renal function phenotypes in T2DM patients, such as DKD, eGFR or creatinine/albumin ration [52,53,54,55,56,57,58,59,60,61]. The *ENPP1* gene is located in the 6q22.q23 region (http://www.ncbi.nlm.nih.gov/gene). Therefore, although some studies reported associations between polymorphic markers on chromosome 6q with DKD phenotypes [53,56,59], the reported closest region to 6q22-q23 was an association of a marker on 6q22.31 with survival on dialysis rates in African-Americans T2DM patients [61]. Moreover, Mooyaart *et al*. [62] performed a meta-analysis to assess the pooled effects of several genetic variants that have reproducibly been associated with DKD in previous studies. Their search identified 34 replicated genetic variants and, of these, 21 remained associated with DKD in the meta-analysis. Importantly, the K121Q polymorphism was not included among the 34 identified genetic variants since the results regarding this polymorphism were not constantly replicated in different populations.

The specific mechanisms that explain the association between the Q allele and risk for DKD are not known [7]. However, it is biological plausible that ENPP1 has a role in kidney tissue injury since it is acknowledged that *ENPP1* gene is expressed in both kidney mesangial and endothelial cells [63], and these cells show progressive pathological changes during the progression from normoalbuminury to overt DKD [7,18]. The Q allele interacts more strongly with the insulin receptor than the K allele, decreasing the autophosphorylation of this receptor [12]. The Q allele carriers seem to exhibit worse IR and hyperinsulinemia than subjects with the KK genotype [64]. Hyperinsulinemia might increase sodium resorption in the kidneys, causing augmented sympathetic-adrenergic activity, volume expansion, and increased expression of the angiotensin type II receptor, impairing peripheral vasodilatation [65]. Reduced vasodilatation as well as increase volume might predispose to arterial hypertension, a well known risk factor for DKD [7,65,66].

Meta-analysis has been regarded as a powerful tool for pooling data from several studies, which could overcome the problem of small sample numbers as well as insufficient statistical power of genetic association studies of complex diseases [26]. Of note, the present meta-analysis had an 80% power (α = 0.05) to detect an OR ≥1.35. Nevertheless, the results of the present meta-analysis should be interpreted within the context of a few limitations. Meta-analyses can be prone to publication bias, and although we made every effort to find unpublished results, we cannot be sure if small negative studies were overlooked. One of the studies identified [23] was not included in our meta-analysis because its control group was constituted of healthy subjects without DKD, and our inclusion criteria were restricted to studies that comprised control subjects with DM but without any degree of DKD. Keene *et al*. [23] did not observe any association between the K121Q polymorphism and ESRD in T2DM patients from an African-American population. In addition, heterogeneity could be a significant problem when interpreting the findings of any meta-analysis. In short, within these limitations, our data seem to be robust, since we did not detect any significant inter-study heterogeneity in any of the genetic inheritance models analyzed.

In conclusion, our results indicate that the *ENPP1* K121Q polymorphism is associated with risk of DKD in European and Asian populations. Since only small sample sizes could be obtained for analyses stratified by ethnicity, further studies with larger sample sizes are needed to confirm the effect possibly played by ENPP1 in the pathogenesis of DKD and related features.

## Supporting Information

S1 PRISMA Checklist (DOC)

S1 Table Meta-analysis on genetic association studies checklist. (DOCX)

S2 Table Characteristics of the studies included in the meta-analysis. Legend: NA: not evaluated. DKD: Diabetic Kidney Disease. 1: Urinary albumin excretion (UAE) (mg/24h): 796(16-14545). 2: Albumin Creatinine Ratio (ACR): 250 mg/g (men) or 355 mg/g (women). 3: Microalbuminuria: UAE 20–199 μg/min or Proteinuria: UAE ≥200 μg/min. 4: Microalbuminuria: UAE 20–199 μg/min or Proteinuria: UAE ≥200 μg/min. 5: Positive dipstick test for protein; Macroalbuminuria: two tests of spot urinary albumin >300 mg/mg of creatinine. 6: ACR >300 μg/mg; Blood Urea Nitrogen >20 mg/dl or Creatinine >1.7 mg/dl.
(DOC)

S3 Table Genotype and allele distributions of the *ENPP1* K121Q polymorphism in patients with diabetic kidney disease and control subjects. Legend: ^a^ African descendants; ^b^ European descendants.
(DOC)
