# Supplementary material for: Association between the ENPP1 K121Q Polymorphism and Risk of Diabetic Kidney Disease: A Systematic Review and Meta-Analysis
Source: PLoS One. 2015 Mar 20;10(3):e0118416. doi: 10.1371/journal.pone.0118416 (PMC4368055; doi:10.1371/journal.pone.0118416)
Supplement: S2 Table — Legend: NA: not evaluated. DKD: Diabetic Kidney Disease. 1: Urinary albumin excretion (UAE) (mg/24h): 796(16-14545). 2: Albumin Creatinine Ratio (ACR): 250 mg/g (men) or 355 mg/g (women). 3: Microalbuminuria: UAE 20–199 μg/min or Proteinuria: UAE ≥200 μg/min. 4: Microalbuminuria: UAE 20–199 μg/min or Proteinuria: UAE ≥200 μg/min. 5: Positive dipstick test for protein; Macroalbuminuria: two tests of spot urinary albumin >300 mg/mg of creatinine. 6: ACR >300 μg/mg; Blood Urea Nitrogen >20 mg/dl or Creatinine >1.7 mg/dl. (DOC) [file pone.0118416.s003.doc]

**Table S2.** Characteristics of the studies included in the meta-analysis.

| **Authors [Ref.]** | **Year** | **Method** | **Ethnicity** | ***n*** | **Age (years)** | **Sex (% male)** | **Diabetes duration (years)** | **Creatinine (µmol/L)** | **DKD classification** |
| --- | --- | --- | --- | --- | --- | --- | --- | --- | --- |
| Tarnow et al*.* [] | 2001 | Case | European | 199 | 40.9 | 61.3 | 26.5 (8-54) | 103 (54-684) | 1 |
|  |  | Control | European | 192 | 42.7 | 61.5 | 25.5 (13-55) | 76 (40-116) |  |
| Canani et al.*.* [] | 2002 | Case | European | 352 | 36.5 | 52.75 | 25.5 | 1.2 +/- 1.5 | 2 |
|  |  | Control | European | 307 | 36.0 | 49.2 | 24.0 | 0.9 |  |
| Leitão et al.*.* ^a^ [] | 2008 | Case | African | 73 | NA | NA | NA | NA | 3 |
|  |  | Control | African | 124 | NA | NA | NA | NA |  |
| Leitão et al. ^b^ [] | 2008 | Case | European | 351 | NA | NA | NA | NA | 4 |
|  |  | Control | European | 479 | NA | NA | NA | NA |  |
| Wu et al. [19] | 2009 | Case | Asian | 216 | 58.0 | 54.6 | 15.23 | NA | 6 |
|  |  | Control | Asian | 178 | 56.5 | 43.8 | 12.98 | NA |  |
| De Cosmo et al. [20] | 2009 | Case | European | 200 | NA | NA | NA | NA | 2 |
|  |  | Control | European | 484 | NA | NA | NA | NA |  |
| Lin et al. [] | 2011 | Case | Asian | 215 | 58.2 | 54.8 | NA | NA | 5 |
|  |  | Control | Asian | 201 | 56.5 | 43.8 | NA | NA |  |

NA: not evaluated. DKD: Diabetic Kidney Disease. 1: Urinary albumin excretion (UAE) (mg/24h): 796(16-14545). 2: Albumin Creatinine Ratio (ACR): 250 mg/g (men) or 355 mg/g (women). 3: Microalbuminuria: UAE 20-199 μg/min or Proteinuria: UAE ≥200 μg/min. 4: Microalbuminuria: UAE 20-199 μg/min or Proteinuria: UAE ≥200 μg/min. 5: Positive dipstick test for protein; Macroalbuminuria: two tests of spot urinary albumin >300 mg/mg of creatinine. 6: ACR >300 μg/mg; Blood Urea Nitrogen >20 mg/dl or Creatinine >1.7 mg/dl.
