# Supplementary material for: Association between the ENPP1 K121Q Polymorphism and Risk of Diabetic Kidney Disease: A Systematic Review and Meta-Analysis
Source: PLoS One. 2015 Mar 20;10(3):e0118416. doi: 10.1371/journal.pone.0118416 (PMC4368055; doi:10.1371/journal.pone.0118416)
Supplement: S3 Table — Legend: a African descendants; b European descendants. (DOC) [file pone.0118416.s004.doc]

**Table S3.** Genotype and allele distributions of the *ENPP1* K121Q polymorphism in patients with diabetic kidney disease and control subjects.

| ***ENPP1-K121Q*** | | | **Cases (n) by total and genotype** | | | | **Controls (n) by total and genotype** | | | | **C allele frequency (%)** | | |
| --- | --- | --- | --- | --- | --- | --- | --- | --- | --- | --- | --- | --- | --- |
| Reference | Year | Ethnicity | Total | A/A | A/C | C/C | Total | A/A | A/C | C/C | Cases | Controls | OR (95% CI)^a^ |
| Tarnow et al. [] | 2001 | European | 199 | 141 | 52 | 6 | 192 | 138 | 45 | 7 | 16.1 | 15.4 | 1.05 (0.72 – 1.55) |
| Canani et al. [] | 2002 | European | 352 | 240 | 102 | 10 | 307 | 241 | 59 | 7 | 17.3 | 11.9 | 1.55 (1.14 – 2.12) |
| Leitao et al. [] ^a^ | 2008 | African | 73 | 19 | 35 | 19 | 124 | 32 | 60 | 32 | 50.0 | 50.0 | 1.00 (0.67 – 1.50) |
| Leitao et al. [] ^b^ | 2008 | European | 351 | 208 | 120 | 23 | 479 | 312 | 156 | 11 | 23.7 | 18.6 | 1.36 (1.07 – 1.72) |
| Wu et al. [19] | 2009 | Asian | 216 | 116 | 79 | 20 | 178 | 122 | 46 | 10 | 27.6 | 18.5 | 1.67 (1.18 – 2.34) |
| De Cosmo et al. [20] | 2009 | European | 200 | 139 | 51 | 10 | 484 | 342 | 126 | 16 | 18.0 | 16.0 | 1.11 (0.81 – 1.51) |
| Lin et al*.* [] | 2011 | Asian | 215 | 116 | 79 | 20 | 201 | 140 | 50 | 11 | 27.7 | 17.9 | 1.75 (1.26 – 2.44) |

^a^ African descendants; ^b^ European descendants.
